# *In vitro* propagation of *Gentiana scabra* Bunge *–* an important medicinal plant in the Chinese system of medicines

**DOI:** 10.1186/s40529-014-0056-4

**Published:** 2014-07-24

**Authors:** Shih-Hung Huang, Dinesh Chandra Agrawal, Fang-Sheng Wu, Hsin-Sheng Tsay

**Affiliations:** 1 Department of Applied Chemistry, Chaoyang University of Technology, Taichung, Taiwan; 2 Department of Agronomy, National Chung-Hsing University, Taichung, Taiwan; 3 Department of Biology, Virginia Commonwealth University, Richmond, 23284-2012 VA USA

**Keywords:** Gentiana scabra, Long dan cao, Medicinal plant, Micropropagation, Ventilation closure

## Abstract

**Background:**

*Gentiana scabra* Bunge commonly known as ‘Long dan cao’ in China has been used in traditional Chinese medicines for more than 2000 years. Dry roots and rhizome of the herb have been used for the treatment of inflammation, anorexia, indigestion and gastric infections. Iridoids and secoiridoids are the main bioactive compounds which attribute to the pharmacological properties of this plant. The species is difficult to mass propagate by seed due to the low percentage of germination and limited dormancy period. Wild populations in some locations are considered to be in the endangered category due to over exploitation.

**Results:**

In the present study, we report an efficient micropropagation system. Shoot apices of six weeks old *in vitro* grown *G. scabra* plants were used as explants for the *in vitro* propagation. Induction of multiple shoots (9.1/explant) was achieved on the culture of shoot apices on half strength Murashige and Skoog’s basal medium (MSBM) containing 2.0 mg/L^−1^ 6-benzylaminopurine (BA), 3% sucrose and 0.9% Difco agar. *In vitro* shoots induced profuse rooting on half strength of MSBM supplemented with 0.1 mg/L^−1^ 1-naphthaleneacetic acid (NAA), 3% sucrose and 0.3% gelrite. A two-stage ventilation closure procedure during the *in vitro* culture, and transparent sachet technique enhanced the survival rate of *G. scabra* plantlets to 96% in the greenhouse. Tissue culture plants flowered after 5 months of transfer to pots.

**Conclusions:**

A simple and an efficient *in vitro* propagation protocol of *Gentiana scabra* Bunge by optimizing the medium composition and ventilation closure treatments has been developed. The protocol can be very useful in germplasm conservation and commercial cultivation of *G. scabra* plants.

**Electronic supplementary material:**

The online version of this article (doi:10.1186/s40529-014-0056-4) contains supplementary material, which is available to authorized users.

## Background

For centuries, plants have been used as a prime natural source of alternative medicines all over the world. This old tradition of medicinal plant application has turned into a highly profitable business in the global market, resulting in the release of a large number of herbal products. There has been an ever expanding market of herbs and herbal based medicinal preparations all over the world. *Gentiana* is one of the highly important groups of medicinal plants. The genus *Gentiana* belonging to the family Gentianaceae contains over 400 species widely distributed in alpine habitats in temperate regions of Asia, Europe, and America (Shimada et al*.*[2009]). *Gentiana scabra* Bunge, a perennial herb grows in meadows, hillsides, forest edges and shrubs in North, East and Northeast China (Han and Wang [1993]). *Gentiana* spp. have extensive medicinal values including antiinflammatory, analgesic, antirheumatic, antipyretic, diuretic and hypoglycemic properties (Chen et al. [2008]; Sezik et al. [2005]; Wani et al. [2011]). Dried roots of *Gentiana scabra* Bunge commonly known as ‘Long dan cao’ in traditional Chinese herbal medicines have been used in the treatment of inflammation, anorexia, indigestion and gastric infections for over 2000 years (Tang and Eisenbrand [1992]). Also, dried roots and rootstcoks have been used to eliminate damp-heat and quench the fire of the liver and gall bladder (Annonymous [2010]). *Gentiana* spp. contain major active compounds like gentiopicroside, swertiamarin and loganic acid. Chemical investigation of root extract of *Gentiana* spp. resulted in isolation of a series of iridoids, secoiridoids, xanthones and xanthone glycosides (Aberham et al. [2011]). *Gentiana* spp. contain some of the most bitter compounds known and is used as a scientific basis for measuring bitterness. In addition to its anti-microbial and anti-inflammatory effects, gentiopicroside in *G. scabra* has been shown to inhibit liver dysfunction, and promote gastric acid secretion, making it a popular ingredient in Chinese herbal medicine (Kim et al. [2009]).

Due to the extensive medicinal use, wild *G. Scabra* plants are in huge demand resulting in its over-exploitation and decline in wild populations. In Liaoning Province of China, the species has been listed in the protected plants and considered to be under the endangered category (Li [1992]). Commercial propagation of *G. scabra* through seed has constraints due to shorter seed dormancy period and lower rates of seed germination (Seong et al. [1995]; Son et al. [1999]; Wen and Yang [2010]). The low survival rate (20% to 53%) of *in vitro* plantlets of *G. scabra* in greenhouse poses a serious challenge to growers’ commercialization process (Personal communications with several growers in Taiwan). Therefore, the main objective of the study was to develop a simple and an efficient *in vitro* propagation method of *G. scabra* with a high survival rate of plantlets.

## Methods

### Plant material

*In vitro* plants of *G. scabra* were obtained from the Taiwan Sugar Corporation, Taiwan, and were used as the source material for the present study. Shoot apices (1.0 cm) excised from the six weeks old *in vitro* plants were used as explants.

### Induction of multiple shoots

For induction of multiple shoots, explants were cultured on Murashige and Skoog’s (Murashige and Skoog [1962]) salts and vitamins, hereinafter referred as MS basal medium (MSBM). A range of 6-benzylaminopurine (BA) (0.1, 0.5, 1.0 and 2 mg/L^−1^) and Kinetin (Kin) concentrations (0.1, 0.5 and 1.0 mg/L^−1^), 3% sucrose, 0.9% Difco Bacto agar (Sigma-Aldrich, St. Louis, MO) were supplemented to the MSBM. The pH of all the media was adjusted to 5.7 ± 0.1, prior addition of agar and before autoclaving for 15 min under 1.05 kg/cm at 121°C.

### Culture vessel and culture conditions

**Figure 1:**
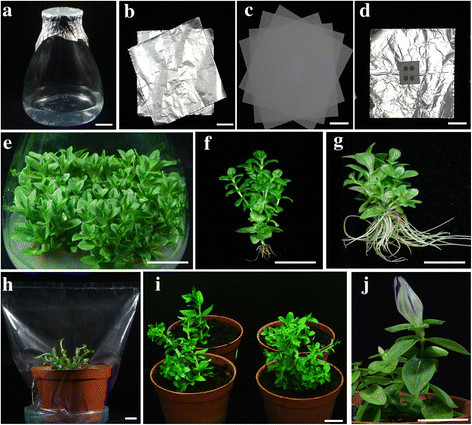
***In vitro*** **propagation of** ***Gentiana scabra*** **Bunge. a**: Orchid culture flask, **b**: Two layers of aluminium foil (2AF), **c**: Four layers of dispense papers; **d**: Two layers of aluminium foils with four holes (diameter 0.5 cm) punched in the center of foil and covered by 2 layers of air permeable 3 M Nexcare tape (4H2AF), **e**: Multiple shoots, **f**: Source culture obtained from Taiwan Sugar Corporation, Taiwan, **g**: Rooted shoots (medium with 0.1 mg/L^−1^ NAA), **h**: A potted plant covered with plastic sachet, **i**: Potted plants (acclimatized), **j**: Plant in the flowering stage in the University’s greenhouse. Scale bar = 2 cm

Orchid culture flask, a modified Erlenmeyer-style transparent glass flask with a 3.5 cm opening on a nearly neckless brim, a base of 9 cm diameter, a height of 12 cm, and 650 ml capacity (Figure 1a) was used as the culture vessel. Each flask contained 100 ml of culture medium. All the cultures were incubated at 25 ± 2°C with a 16/8 h (day/night) cycle under a light intensity of 38 μmol/m^2^/s provided by while fluorescent tubes.

### Induction of rooting in in vitro shoots

The shoots induced from shoot apexes were cultured on MSBM supplemented with two auxins, NAA (0.1, 0.5 and 1.0 mg/L^−1^) or IBA (0.1, 0.5 and 1.0 mg/L^−1^), 3% sucrose and 0.3% gelrite. After 8 weeks of incubation, rooted shoots were carefully taken out of the culture vessels. Their fresh weights, number of roots and shoots, length of roots and shoots were recorded.

### Optimization of ventilation closures

Different ventilation closure treatments were tested by enclosing the flask openings with the following materials: (**a**) 2 layers of aluminium foil (AF) (Figure 1b) (9.5 × 9.5 cm, 0.046 mm thick; Reynolds Consumer Products, Alcoa Inc., Richmond, Virginia, USA; (**b**) 2–4 layers of dispense papers (DP) (Figure 1c) (9.5 × 9.5 cm; 0.046 mm thick; gas flow 0.5 ml/s; made from soft and hardwood fiber at 50:50; Cheng Long Corporation, Taiwan; (**c**) 2 layers of aluminum foil with four holes (diameter 0.5 cm) punched in the center of foil covered by 2 layers of air permeable 3 M Nexcare tape (Figure 1d) (3 M Corp., St. Paul, MN, USA). There were total 9 ventilation closure treatments grouped into two sets: (1) One-stage, where culture vessels were closed with 2 layers of aluminum foil or 2,3,4 layers of dispense paper and the total incubation period of 8 weeks; (2) Two-stage, where each culture vessel was first closed with two layers of AF for 1, 2, 3 and 4 weeks followed by replacement of the AF with 4DP in a laminar flow cabinet and continuation of incubation for further 7, 6, 5 and 4 weeks, i.e. total 8 weeks. According to the duration of incubation periods and the container closure materials, the treatment codes were designated as listed in Table 1: **(1)** 2 layers of aluminum foil (AF) and incubation for 8 weeks (2AF8wk), **(2)** 2 layers of dispense paper (DP) for 8 weeks (2DP8wk), (**3**) 3 layers of DP for 8 weeks (3DP8wk), **(4)** 4 layers of DP for 8 weeks (4DP8wk), **(5)** 2 layers of AF with four holes punched in the center of foil covered by 2 layers of air permeable tape and incubation for 8 weeks (4H2AF8wk) (**6**) 2 layers of AF for one week then AF replaced by 4 DP for the next 7 weeks (2AF1wk/4DP7wk), (**7**) 2 layers of AF for 2 weeks then AF replaced by 4 DP for the next 6 weeks (2AF2wk/4DP6wk), (**8**) 2 layers of AF for 3 weeks then AF replaced by 4 DP for the next 5 weeks (2AF3wk/4DP5wk), (**9**) 2 layers of AF for 4 weeks then AF replaced by 4 DP for the next 4 weeks (2AF4wk/4DP4wk). Thus, for all the cultures the total incubation period was kept constant, i.e. 8 weeks. Medium composition for each ventilation closure treatment consisted of half strength MSBM supplemented with 0.1 mg/L^−1^ NAA, 3% sucrose and 0.3% gelrite.

**Table 1:** **Influence of ventilation closure types on root/shoot growth parameters and** ***ex vitro*** **plant survival in** ***G. scabra*** **Bunge** ^**X**^

Ventilation closure treatment code		No. of roots^Z^	Root length^Z^(cm)	Shoot length^Z^(cm)	Fresh weight^YZ^(g)	Plant survival rate^Z^(%)
One-stage	2AF8wk	20.2 ± 9.0 a	4.58 ± 1.16 a	2.23 ± 0.69 a	1.09 ± 0.46 a	94.44 ± 4.30 a
	2DP8wk	12.4 ± 9.3 b	3.03 ± 1.18 c	1.28 ± 0.32 c	0.77 ± 0.42 c	79.13 ± 13.93 b
	3DP8wk	13.0 ± 7.7 b	3.02 ± 0.89 c	1.43 ± 0.31 bc	0.78 ± 0.47 c	89.92 ± 8.36 ab
	4DP8wk	18.0 ± 10.2 a	3.78 ± 1.01 b	1.51 ± 0.40 b	0.94 ± 0.33 b	91.67 ± 6.01 ab
	4H2AF8wk	15.9 ± 4.98 a	4.25 ± 0.92 b	1.93 ± 0.88 a	0.93 ± 0.45 a	91.11 ± 8.31 ab
Two-stage	2AF1wk/4DP7wk	23.0 ± 9.7 b^Z^	4.44 ± 1.12 c^Z^	1.89 ± 0.42 b^Z^	1.14 ± 0.32 b^Z^	91.67 ± 2.89 a^Z^
	2AF2wk/4DP6wk	29.6 ± 12.0 a	5.78 ± 1.13 a	2.31 ± 0.59 a	1.29 ± 0.40 ab	90.00 ± 3.33 a
	2AF3wk/4DP5wk	28.6 ± 13.5 a	5.15 ± 1.20 b	2.48 ± 0.74 a	1.23 ± 0.46 b	93.33 ± 4.67 a
	2AF4wk/4DP4wk	33.7 ± 12.5 a	5.89 ± 0.92 a	2.35 ± 0.51 a	1.41 ± 0.45 a	96.25 ± 3.60 a

^X^Medium: Half strength MSBM, 0.1 mg/L^−1^ NAA, 3% sucrose and 0.3% gelrite, incubation period of 8 weeks.

^Y^Fresh weight of rooted shoot (plantlet).

^Z^Means followed by the same letter of a column are not significantly different at 5% probability (*p = 0.05*) level by least significant difference (LSD) test.

### Acclimatization of plantlets

After 8 weeks of incubation under the ventilation closure conditions described above, the plantlets were carefully taken out of culture vessels and rinsed gently with running tap water to remove traces of agar. The plantlets were then blotted dry on paper towel and their fresh weights, number of shoots and roots, length of roots and shoots were recorded. Thereafter, plantlets were briefly treated with 0.1% Benlate (a systemic fungicide) solution (DuPont, Wilmington, DE) and transplanted into plastic pots containing a mixture of peatmoss:perlite:vermiculite (2:1:1 v/v). For acclimatization, each potted plant was covered with a transparent polyethylene sachet (Figure 1h). After one week, a small hole was made in a corner of each sachet, and another hole was made in the opposite corner after 2 weeks. The top corners on both sides of each sachet were cut open after 3 weeks, and sachets were completely removed after 4 weeks. Thereafter, these were shifted to the University’s greenhouse. The plants were irrigated every day with tap water. The plant survival against each ventilation closure treatment was recorded after 2 months.

### Statistical analysis

Data were analyzed statistically by using Statistical Analysis System SAS 9.1 for ANOVA and the least significant difference (LSD) tested at 5% probability level (*p ≥* 0.05). All experiments were repeated minimum three times. There were minimum 30 replicates under each treatment.

## Results and discussion

### Multiple shoot induction

In our initial experiments on different strengths (1X, 1/2X and 1/4X) of MSBM medium, it was observed that 1/2X MSBM medium resulted in the better shoot/root growth in comparison to 1X or 1/4X strengths (data not shown), hence, 1/2X MSBM medium was used for all the experiments. Between the two cytoknins, BA was more effective and induced a higher number of shoots per explant compared to Kin (Table 2) (Figure 1e). The maximum average number of multiple shoots (9.1 shoots/explant) could be induced in shoot apices on half strength MSBM supplemented with 2.0 mgL^−1^ BA, 3% sucrose and 0.9% agar, followed by 8.8 shoots/explant on medium with 1.0 mgL^−1^ BA. There was an inverse correlation between concentrations of both BA and Kin and lengths of induced shoots. Average shoot elongation was higher (2.82 and 3.56 cm) at lower concentrations of BA (0.1 mgL^−1^) and Kin (0.1 mgL^−1^), respectively (Table 2). Similar to the present study, BAP was found to be the most effective cytokinin in several plant species, i.e. *Gossypium* (Agrawal et al. [1997]), *Salix* (Agrawal and Gebhardt [1994]), *Pisum* (Jackson and Hobbs [1990]), *Phaseolus* (McClean and Grafton [1989]) and *Glycine* (Cheng et al. [1980]), indicating a particular cytokinin preference of certain tissues for the induction of multiple shoots. In an earlier report on *G. scabra*, induction of multiple shoots in axillary buds of was achieved on MS medium supplemented with gibberlliin A_3_ (GA_3_) and BA (1 mg/L^−1^ each) (Yamada et al. [1991]). In another study, adventitious buds were induced in stem callus of *G. scabra* cultured on MS medium supplemented with 1.2 mgL^1^AgNO_3_ + 0.6 mgL^1^ BA + 0.1 mgL^1^ NAA (Wen and Yang [2010]).

**Table 2:** **Induction of multiple shoots in shoot apices of** ***Gentiana scabra*** **Bunge**

Cytokinin^X^(mg/L^−1^)		No. of shoots^Y^	Shoot length^Y^(cm)	Fresh weight of rooted shoot^Y^(g)
BA	0	5.6 ± 0.8 c	4.82 ± 0.49 a	0.50 ± 0.15 d
	0.1	7.1 ± 1.0 b	2.82 ± 0.27 d	0.55 ± 0.08 bcd
	0.5	7.4 ± 1.4 b	2.43 ± 0.24 e	0.55 ± 0.17 bcd
	1.0	8.8 ± 2.8 a	2.23 ± 0.43 e	0.53 ± 0.10 cd
	2.0	9.1 ± 1.8 a	1.92 ± 0.42 f	0.53 ± 0.17 cd
Kin	0.1	5.5 ± 0.9 c	3.56 ± 0.31 b	0.59 ± 0.07 abc
	0.5	6.0 ± 0.8 c	3.31 ± 0.17 c	0.60 ± 0.09 ab
	1.0	5.7 ± 1.1 c	3.13 ± 0.26 c	0.60 ± 0.15 a

– Influence of BA and Kin.

^X^Medium – half strength MSBM, 3% sucrose, 0.9% agar, incubation period 6 weeks.

^Y^Means followed by the same letter of a column are not significantly different.

at 5% probability (*p = 0.05*) level by least significant difference (LSD) test.

### Induction of rooting in in vitro shoots

On culture of *in vitro* derived shoots on 1/2 strength MSBM medium supplemented with a range of concentrations of two auxins (NAA and IBA) for 8 weeks, it was observed that the lower concentrations of NAA or IBA resulted in a higher number as well as better root/shoot growth. NAA at 0.1 mg/L^−1^ induced the greater number of roots (37.2) and fresh weight (1.55 g) in comparison to IBA at 0.1 mg/L^−1^ which induced a lesser number of roots (25.8) and lesser fresh weight (1.20 g) (Table 3). Higher concentrations of IBA and NAA (1.0 mg/L^−1^) reduced the number and length of roots as compared to 0.1 mg/L^−1^ concentration. In contrast to culture (Figure 1f) obtained from Taiwan Sugar Corporation, roots induced in the MSBM medium supplemented with NAA 0.1 mg/L^−1^ were multifold and robust (Figure 1g). A supplement of 1.0 mg/L^−1^NAA in the MSBM stimulated callus induction, a trait undesirable for health of roots/shoots and *ex vitro* survival of plantlets.

**Table 3:** **Induction of rooting in** ***in vitro*** **shoots of** ***G. scabra*** **Bunge - Influence of auxins on root/shoot growth parameters** ^**X**^

Auxin(mg/L^−1^)		No. of roots^Y^	Root length^Y^(cm)	Shoot length^Y^(cm)	Fresh weight^Y,Z^(g)
NAA	0	26.5 ± 7.3 b	5.07 ± 1.24 b	3.89 ± 1.39 a	0.96 ± 0.34 e
	0.1	37.2 ± 12.1 a	5.83 ± 1.03 a	3.06 ± 0.68 b	1.55 ± 0.38 a
	0.5	33.2 ± 17.5 b	4.51 ± 1.14 c	2.65 ± 1.17 c	1.44 ± 0.44 cb
	1.0	25.3 ± 22.1 b	2.96 ± 1.33 d	1.58 ± 0.96 d	1.32 ± 0.52 ab
IBA	0.1	25.8 ± 6.3 b	5.44 ± 0.82 ab	3.33 ± 0.91 b	1.20 ± 0.29 cd
	0.5	16.2 ± 7.5 c	4.55 ± 1.47 c	2.37 ± 0.80 c	1.17 ± 0.39 de
	1.0	12.1 ± 5.9 c	4.15 ± 1.30 c	2.04 ± 0.68 c	1.15 ± 0.36 d

^X^Medium: half strength MSBM, 3% sucrose and 0.3% gelrite, Incubation period of 8 weeks.

^Y^Means followed by the same letter of a column are not significantly different at the 5% probability (*p = 0.05*) level by least significant difference (LSD) test.

^Z^Fresh weight of rooted shoot.

Auxins are known to affect both root and shoot growth parameters and play an important role particularly in root development. Different plant species respond differently to auxins for the induction of rooting. The promotory effect of a lower salt concentration of MS on *in vitro* rooting of shoots has been reported for *Gossypium* (Agrawal et al. [1997]), *Philodendron* spp. (Maene and Debergh [1985]). Some plant species, even do not require any auxin supplemental in the medium for rooting (Agrawal and Gebhardt [1994]), hence it is desirable to optimize the type and concentration of an auxin in a micropropagation protocol of a particular plant species. In contrast to our results, induction of rooting in shoots of *Gentiana scabra* was achieved on hormone free MS medium (Yamada et al. [1991]), or on 1/2 MS medium supplemented with 0.3 mgL^1^ NAA + 0.1 mgL^1^ IAA (Wen and Yang [2010]). Akin to the present study, higher concentrations of NAA induced callus in *G. davidii* var. *formosana* (Chueh et al*.*[2001]).

### Optimization of ventilation closures

**Figure. 2:**
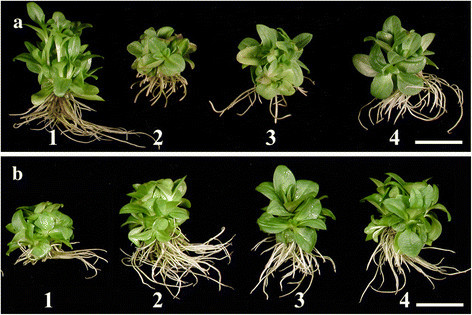
**Influence of ventilation closures on root/shoot growth in** ***G.*** *scabra* Bunge **(a)** One-stage ventilation closure: (1) 2AF8wk-2 layers of aluminum foil (AF) and incubation for 8 weeks, (2) 2DP8wk-2 layers of dispense papers (DP) for 8 weeks, (3) 3DP8wk-3 layers of DP for 8 weeks, (4) 4DP8wk-4 layers of DP for 8 weeks, **(b)** Two-stage ventilation closure: (1) 2AF1wk/4DP7wk-2 layers of AF for one week then AF replaced by 4 DP for the next 7 weeks, (2) 2AF2wk/4DP6wk-2 layers of AF for 2 weeks then AF replaced by 4 DP for the next 6 weeks, (3) 2AF3wk/4DP5wk-2 layers of AF for 3 weeks then AF replaced by 4 DP for the next 5 weeks, and (4) 2AF4wk/4DP4-2 layers of AF for 4 weeks then AF replaced by 4 DP for the next 4 weeks. Scale bar = 2 cm

Between the two sets of ventilation closure treatments, i.e. one-stage and two-stage with a total incubation period of 8 weeks, overall, all the root/shoot growth parameters including the plant survival percentage were higher with two-stage ventilation closure treatments (Table 1). There were significant differences in root/shoot growth parameters among the treatments within the one-stage, and two-stage ventilation closures itself (Figure 2 a1-4 and b1-4). Among the one-stage ventilation closure treatments, the maximum number of roots (20.20), root length (4.58 cm), shoot length (2.23 cm), fresh weight (1.09 g) and plant survival percentage (94.44) was achieved with 2AF8wk, i.e. when culture vessels were closed with 2 layers of aluminum foil and incubation for 8 weeks. In contrast to aluminum foil, there was an overall decrease in root/shoot growth parameters when culture vessels were closed with 2, 3 and 4 dispense papers. Among the three DP treatments, the maximum number of roots (18), root length (3.78 cm), shoot length (1.51 cm), fresh weight (0.94 g) and plant survival percentage (91.67) was achieved with 4DP8wk, i.e. when culture vessels were closed with 4 layers of dispense paper and incubation for 8 weeks. There was no drastic difference in root/shoot growth parameters and plant survival percentage in the ventilation closure treatment 4H2AF8wk, i.e. 2AF with 4 holes punched in the center and covered with air permeable tape. Among the 4 two-stage ventilation closure treatments, the maximum number of roots (33.7), root length (5.89 cm), fresh weight (1.41 g) and plant survival (96%) were obtained with treatment 2AF4wk/4DP4wk, i.e. when culture vessels were closed with 2 AF for 4 weeks and then AF replaced by 4DP for the next 4 weeks (Table 1).

It is essential to close a culture container with some closure material to maintain sterility of the cultures. Different types of container closure materials like micropore, parafilm, and polyvinyl chloride for eggplants (Ribeiro et al. [2009]), plastic films for neem (Rodrigues et al. [2012]) have been used. In our laboratory, aluminum foil and dispense paper have been used successfully to improve ventilation for cultures of *Scrophularia* (Chen et al. [2006a]), and *Bupleurum* (Chen et al. [2006b]). It has been reported that the type of closure affects gaseous exchange, availability of water, micronutrients, and balance of hormones in the culture container (Kataeva et al. [1991]; Lai et al. [2005]; Chen et al. [2006a]; Tsay et al. [2006]). The head space of culture vessels with low ventilation, accumulates various gaseous compounds like ethylene and carbon dioxide (Akhter Zobayed et al. [2001]; Lai et al. [2005]). These undesirable compounds can alter biochemical responses and leaf development of *in vitro* cultured plants (Pierik et al. [2007]) and also affect enzymes involved in oxidative activities (Synková and Pospíšilová [2002]). Some closures cause restriction of gaseous exchange between the container atmosphere and the outside environment (Buddendorf-Joosten and Woltering [1994]), which can result in poor aeration and hyperhydric condition of cultures. Also, growth rate and other physiological and morphological characteristics of plants developed under *in vitro* conditions can be influenced by the physical and chemical micro-environments of culture containers (Walker et al. [1988]). Different species show different requirement with respect to container closures. Hence, it is important to optimize a closure type in a micropropagation protocol of a particular plant species. In the present study, a new approach of combining the foil and dispense paper was used for the *G.* s*cabra* cultures*.* It was observed that aluminum foil, a less air permeable material for first 4 weeks, followed by more air permeable dispense papers for next 4 weeks was an adequate ventilation treatment for optimum root/shoot growth and subsequent survival of *G.* s*cabra* plantlets.

### Influence of ventilation closures on survival of plantlets, and acclimatization procedure

Between the one-stage and two-stage ventilation closure treatments, the overall survival percentages were higher (90–96) in the two-stage treatments (Table 1). The maximum survival percentage (96) was recorded with the two-stage treatment 2AF4wk/4DP4wk, i.e. culture vessel closed with 2 layers of aluminum foil for first 4 weeks and then AF replaced with 4 layers of disense paper. Covering of plants with transparent polyethylene sachets for the first 4 weeks and its gradual exposure to the ambient conditions was supportive of acclimatization process and gave rise to higher plantlet survival percentages. Tissue culture plants flowered (Figure 1j) in the greenhouse after 5 months of their transfer to pots.

## Conclusions

In the present study, we have developed a simple and an efficient *in vitro* propagation protocol of *Gentiana scabra* Bunge by optimizing the medium composition and ventilation closure treatments. The developed protocol may be very useful in micropropagation, germplasm conservation and commercial cultivation of *G. scabra* plants.

## Authors’ original submitted files for images

Below are the links to the authors’ original submitted files for images. Authors’ original file for figure 1 Authors’ original file for figure 2

## Abbreviations

BA: 6-benzylaminopurine
IBA: Indole-3-butyric acid
MSBM: Murashige and Skoog's basal medium (MS salts and vitamins)
NAA: 1-naphthaleneacetic acid
