# Supplementary figures and images for: In vitro propagation of Gentiana scabra Bunge – an important medicinal plant in the Chinese system of medicines
Source: Bot Stud. 2014 Jul 24;55:56. doi: 10.1186/s40529-014-0056-4 (PMC5430348; doi:10.1186/s40529-014-0056-4)

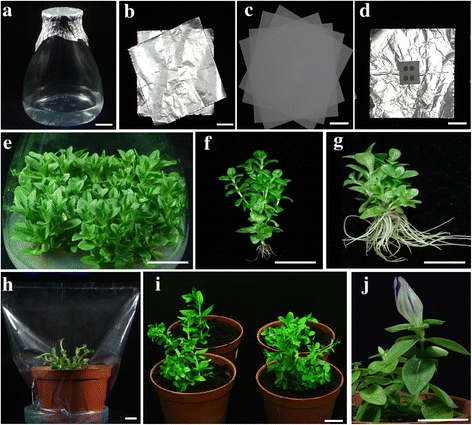

Supplement: Supplementary file 1 — Authors’ original file for figure 1 [file 40529_2014_9056_MOESM1_ESM.gif]

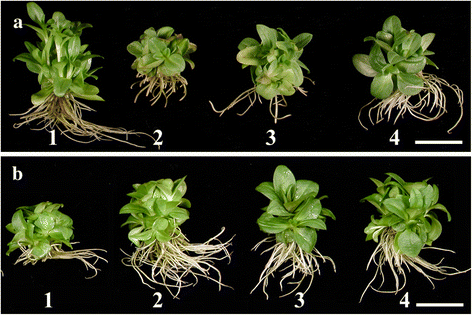

Supplement: Supplementary file 2 — Authors’ original file for figure 2 [file 40529_2014_9056_MOESM2_ESM.gif]
